# Assessment of the vasoactive-inotropic score in prognostic evaluation of critical patients following noncardiac surgery: a retrospective, observational study

**DOI:** 10.1186/s12871-026-03724-z

**Published:** 2026-03-09

**Authors:** Rongjie Jiang, Xiaodong Song, Huan Ma, Shuhe Li, Ka Yin Lui, Xiaoguang Hu, Yanping Zhu, Changjie Cai, Zhaoxia Tang

**Affiliations:** 1 Department of Critical Care Medicine, The First Affiliated Hospital of Sun Yat-Sen University, Guangzhou, 510080 China; 2 Department of Critical Care Medicine, The First People’s Hospital of Foshan, Foshan, 528000 China; 3 University of Exeter Medical School, University of Exeter, Heavitree Road, Exeter, Devon EX1 2LU UK

**Keywords:** Vasoactive-Inotropic Score, Noncardiac surgery, Sequential organ failure assessment, Prognosis, Intensive care unit

## Abstract

**Objective:**

To explore the value of the Vasoactive-Inotropic Score (VIS) in evaluating the prognosis of patients admitted to the ICU after noncardiac surgical operations.

**Methods:**

A retrospective analysis was conducted on 3,365 post-noncardiac surgical patients admitted to the Department of Critical Care Medicine, The First Affiliated Hospital of Sun Yat-sen University, from January 2018 to June 2022. The VIS was calculated based on the maximum and average doses of vasoactive and inotropic agents administered within the first 24 h after noncardiac surgery. Patients were divided into five groups according to VIS levels: 0–5, 6–15, 16–30, 31–45, and > 45 points.

**Results:**

The primary outcome was 28-day mortality. The mean 24-h maximum VIS of post-noncardiac surgical ICU patients was 1.2, with over half of the patients having a VIS below 5 points. Mortality in patients with higher VIS was significantly higher than in those with lower VIS, with the mortality rates of the five VIS groups being 7.5%, 9.4%, 12.1%, 17.2%, and 33.8%, respectively. Kaplan–Meier survival curves showed that patients with a VIS > 45 had poorer survival rates. The incidence of infection and sepsis increased with higher VIS. Multivariate logistic regression analysis identified a 24-h maximum VIS > 45 was an independent risk factor for 28-day mortality. VIS yielded an AUC of 0.744 for predicting ICU mortality and 0.683 for predicting 28-day mortality. In short-term prognosis assessment, the predictive value of VIS was better than the Sequential Organ Failure Assessment (SOFA) score and serum lactate level.

**Conclusions:**

Adult post-noncardiac surgical ICU patients have a high incidence of infection and poor outcomes. A higher VIS robustly predicts adverse postoperative outcomes, with independent prognostic value in this Chinese cohort. Though not supplanting established tools (e.g., Acute Physiology and Chronic Health Evaluation II [APACHE II], SOFA), VIS serves as a complementary indicator, providing additional real-time hemodynamic insights. Larger prospective studies are needed to verify whether VIS-guided interventions improve patient outcomes.

**Supplementary Information:**

The online version contains supplementary material available at 10.1186/s12871-026-03724-z.

## Background

Inotropic agents and vasopressors are frequently administered in operating rooms and intensive care units for hemodynamic support [1]. Hemodynamic instability often leads to increased mortality; therefore, the dosage of vasoactive agents used is generally correlated with the severity of the disease [2, 3]. A variety of vasoactive-inotropic agents are commonly employed to stabilize hemodynamic conditions, including norepinephrine, epinephrine, dopamine, dobutamine and vasopressin [4]. These vasoactive agents have distinct pharmacology, hemodynamic effects and dosages, often exhibiting significant heterogeneity in clinical practice [5]. High doses of vasoactive agents and inotropes have been shown to exert detrimental effects on organ function, frequently resulting in increased myocardial oxygen consumption, arrhythmias, ischemic perfusion injury and immune-mediated organ damage [6–8].

The Vasoactive-Inotropic Score (VIS), proposed by Gaies in 2010, is a widely used tool for quantitatively assessing the dosage of vasoactive agents [9]. Vascular paralysis is a common phenomenon following cardiac surgery [10]. Initially developed to quantify vasoactive and inotropic support in pediatric patients after cardiac surgery, numerous studies have demonstrated that the VIS is closely associated with adverse outcomes following cardiovascular surgery [11].

However, the VIS has not been fully investigated in the setting of noncardiac surgery. Accumulating evidence have indicated that early postoperative hypotension is associated with adverse postoperative outcomes in noncardiac surgical patients, including myocardial infarction, acute kidney injury, cerebrovascular events, delirium and mortality [12]. Postoperative hypotension may serve as a modifiable risk factor for adverse postoperative outcomes, and vasoactive agents and inotropes constitute a first-line therapeutic intervention for the management of postoperative hypotension [13]. A recent multicenter study suggested that postoperative vasoactive agent administration is closely correlated with postoperative complications and poor prognosis in patients after noncardiac surgery [14]. Nevertheless, this study, along with most other relevant investigations, failed to further elucidate the dosage of vasoactive agents administered and lacked a standardized approach to incorporate different classes of vasoactive agents and inotropes. To date, the optimal hypotension intervention threshold and the optimal vasoactive agent and inotropes burden for ICU-admitted patients following noncardiac surgery remain undefined, and the predictive efficacy of VIS for evaluating the prognosis of noncardiac surgical patients has not been thoroughly explored [15, 16]. The primary objective of this study was to investigate the utilization of vasoactive agents in patients after noncardiac surgery, to assess the predictive value of VIS for the prognosis of patients admitted to the ICU following noncardiac surgery, and to compare its performance with other widely used ICU prognostic scoring systems.

## Methods

### Patients selection

**Fig. 1:**
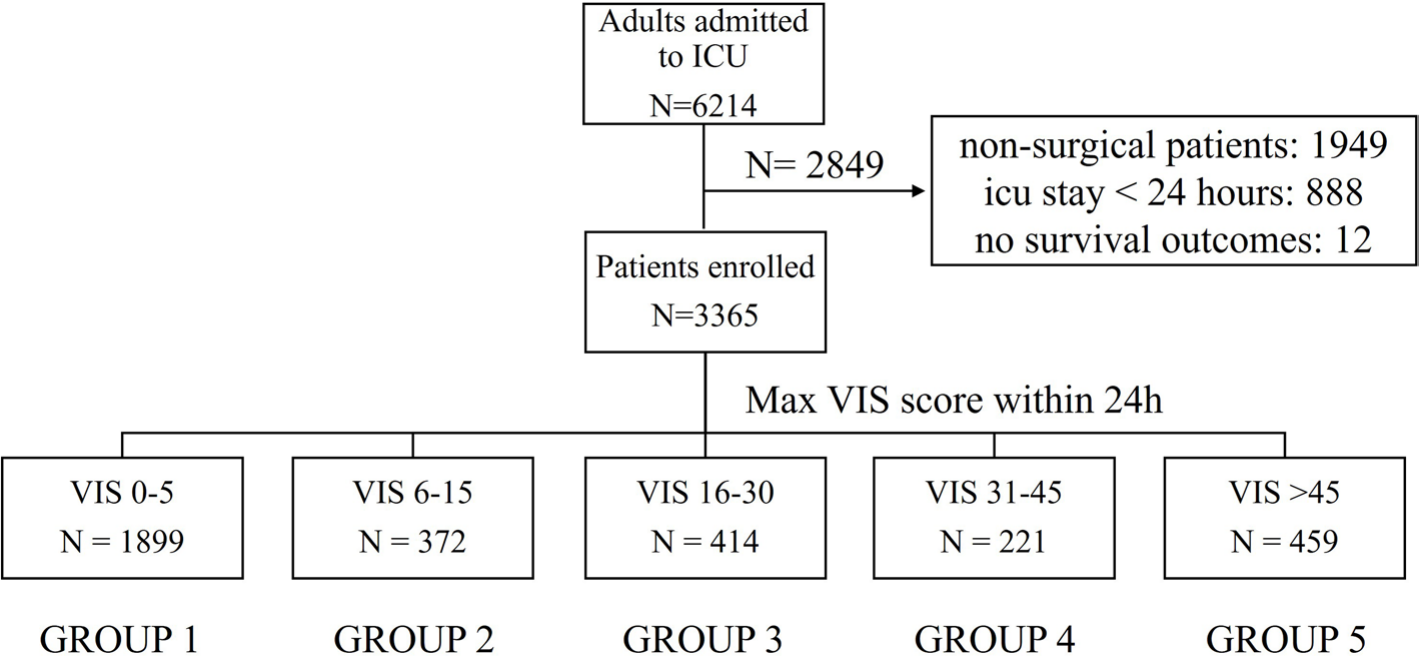
Study participant inclusion flow diagram

We conducted a retrospective cohort study, enrolling all noncardiac surgical patients admitted to the Department of Critical Care Medicine, First Affiliated Hospital of Sun Yat-sen University, from January 2018 to June 2022. The exclusion criteria included no confirmed noncardiac surgical operation performed, lack of data related to survival prognosis, and ICU length of stay less than 24 h. In accordance with prior studies, the study cohort was stratified into five subgroups by 24-h maximum VIS levels: 0–5, 6–15, 16–30, 31–45, and > 4511 (Fig. 1).

### Data extraction

Clinical and demographic data of the enrolled patients were collected, including age, gender, height, weight, types and dosages of vasoactive agents used, Charlson Comorbidity Index (CCI), comorbid underlying comorbidities, surgical approach, vital signs, central venous pressure, fluid management, time of ICU admission and transfer to other departments, date of death, or date of last follow-up. After patients were discharged from the ICU or hospital, prognostic data were obtained via long-term telephone follow-up with patients or their family members. All data were extracted using Structured Query Language (SQL) queries via the pgAdmin4 (version 6.15) interface for PostgreSQL.

### Treatment protocol

All patients admitted to the ICU following noncardiac surgery were managed in accordance with standard ICU clinical protocols. The SOFA score was assessed on ICU admission. Based on the Sepsis-3 definition, the diagnostic criteria for sepsis included an increase in SOFA score by ≥ 2 points due to current infection [17]. For patients presenting with hypotension (defined as a systolic blood pressure < 100 mmHg), fluid responsiveness was evaluated utilizing standardized protocols, including inferior vena cava variability assessment and the passive leg raise test [18]. Patients with fluid responsiveness were prioritized for fluid resuscitation. For those whose hypotension remained uncorrected after adequate fluid resuscitation, vasoactive agents and inotropic drugs were considered, with dynamic adjustment of drug dosages to maintain a mean arterial pressure (MAP) of ≥ 65 mmHg [19].For patients with sepsis, norepinephrine was the first-line agent for blood pressure maintenance. If a norepinephrine dosage exceeding 0.5 μg/kg/min failed to achieve a mean arterial pressure (MAP) of ≥ 65 mmHg, adjunctive vasoactive agents (e.g., metaraminol) were considered for administration [20].

### Vasoactive-Inotropic Score

The types and dosages of vasoactive agents administered were recorded hourly after patients’ admission to the ICU, and the maximum VIS and average VIS within the first 24 h were calculated accordingly. The maximum VIS was defined as the highest dosage of a single vasoactive agent administered within any one hour, or the combined dosage of multiple vasoactive agents that yielded the maximum VIS, during the 24-h period following ICU admission. The average VIS was calculated as the total dosage of vasoactive agents administered within 24 h divided by the effective duration of vasoactive agent administration over the 24-h period. This study adopted the modified VIS proposed by Gaies et al. in 20,109. The VIS was calculated as follows: VIS = dopamine dose (μg/kg/min) + dobutamine dose (μg/kg/min) + 100 × epinephrine dose (μg/kg/min) + 100 × norepinephrine dose (μg/kg/min) + 10,000 × vasopressin dose (U/kg/min) + 10 × milrinone dose (μg/kg/min).

### Outcome measures

The primary outcomes of this study included 28-day all-cause mortality, while the secondary outcomes comprised ICU mortality, in-hospital mortality, ICU length of stay, and hospital length of stay. Additional adverse outcomes, including infection sites during ICU stay and sepsis, were also extracted. The occurrence and anatomical sites of infection were confirmed based on etiological culture results. Sepsis was diagnosed in accordance with the criterion that the SOFA score increased by ≥ 2 points attributable to the current infection.

### Statistical analysis

Quantitative variables are presented as median and interquartile range (IQR), or mean and standard deviation (SD), and compared using the Mann–Whitney U test for two groups or the Kruskal–Wallis H test for multiple groups; categorical data were presented as n (%) and analyzed using the chi-square (χ^2^) test or Fisher's exact test where appropriate. The association between the VIS and mortality in post-noncardiac surgical patients is evaluated using the log-rank test for survival curves. Pearson correlation analysis was conducted to examine the associations between crucial predictors, including APACHE-II, SOFA, VIS max, VIS average, maximum serum lactate level, CVP and Fluid balance. Univariate logistic regression analysis was performed for all observed variables, and those with statistical significance in univariate analysis as well as clinical relevance were included in the subsequent model. The VIS was categorized into five groups (0–5, 6–15, 16–30, 31–45, and > 45 points) and entered into the model as a categorical variable with dummy variables generated (0–5 points as the reference group). Stepwise logistic regression analysis was was performed to identify independent predictors of 28-day mortality, with a total of 11 variables ultimately included in the final model, namely age, gender, APACHE II score, CCI, VIS groups (four dummy variables), sepsis occurrence, and lactate level. Multicollinearity was assessed using the variance inflation factor (VIF), with a VIF value < 5 considered indicative of no significant multicollinearity. Calibration analysis was also performed to examine the reliability of our multivariable regression model. The receiver operating characteristic (ROC) curve and Wald-2 test are used to analyze the value of the VIS and other critical illness assessment models in evaluating the prognosis of post-noncardiac surgical patients. Additionally, internal validation was performed via the bootstrap method with 1000-resamplings to test the stability of the predictive performance of VIS. A two-sided P-value < 0.05 was considered statistically significant. All statistical analyses were performed using R software (version 4.3.2).

## Results

A total of 6,214 post-noncardiac surgical patients admitted to the ICU were observed in the study. After excluding 2,849 patients, 3,365 patients were finally included (seen in Fig. 1). Based on the VIS, they were divided into 5 groups: 0–5 (*n* = 1,899), 6–15 (*n* = 372), 16–30 (*n* = 414), 31–45 (*n* = 221), and > 45 (*n* = 459). Significant intergroup differences were observed in APACHE II score, SOFA score, vital signs, presence of sepsis and lactate level (all *P* < 0.05) (Table 1). Patients with higher VISs had poorer baseline conditions, higher SOFA scores and Higher incidence of acquiring sepsis. Concurrently, ICU mortality, 28-day mortality, and in-hospital mortality increased significantly with elevated VIS. The mean 24-h maximum VIS of the entire cohort was 1.2 points, with more than half of the patients having a VIS < 5 points, indicating no or only low-dose vasoactive-inotropic agent support was required. A slight elevation in VIS was associated with a marked increase in ICU mortality: the ICU mortality rate was 1.3% in patients with a VIS < 5 points, and 2.4% in the 6–15 points group. Patients with high VIS showed significantly increased short-term and long-term mortality. The overall 28-day mortality of all post-noncardiac surgical patients admitted to the ICU was 12.5%, while the 28-day mortality of patients with a VIS > 45 reached 33.8%.

**Table 1:** Baseline characteristics and clinical outcomes of study population

	Total (*n* = 3365)	0–5 (*n* = 1899)	6–15 (*n* = 372)	16–30 (*n* = 414)	31–45 (*n* = 221)	> 45 (*n* = 459)	p
Male, n (%)	2258 (67.1)	1212 (63.8)	248 (66.7)	301 (72.7)	159 (71.9)	338 (73.6)	< 0.001
Age (years)	59 (47, 69)	57 (45, 69)	61 (51, 69)	60 (48, 68)	61 (51, 73)	59 (50, 69)	< 0.001
BMI (kg/m2)	22.5 (20.1, 24.8)	22.4 (20.2, 24.8)	22.1 (19.8, 24.2)	22.7 (20.2, 24.5)	23 (20.3, 25.6)	22.3 (19.9, 25.1)	0.110
Comorbidities, n (%)							
Hypertension	838 (28.4)	490 (30.6)	96 (28.7)	106 (28.0)	57 (26.9)	89 (21.0)	0.003
Diabetes	490 (16.6)	253 (15.8)	69 (20.6)	64 (16.9)	31 (14.6)	73 (17.2)	0.258
Cancer	1490 (50.5)	770 (48.2)	189 (56.4)	195 (51.6)	123 (58.0)	213 (50.2)	0.010
COPD	35 (1.2)	17 (1.1)	5 (1.5)	7 (1.9)	2 (0.9)	4 (0.9)	0.675
CHD	158 (5.4)	74 (4.6)	21 (6.3)	20 (5.3)	21 (9.9)	22 (5.2)	0.028
CKD	186 (6.3)	113 (7.1)	16 (4.8)	14 (3.7)	10 (4.7)	33 (7.8)	0.046
Cirrhosis	453 (15.4)	184 (11.5)	59 (17.6)	77 (20.4)	46 (21.7)	87 (20.5)	< 0.001
CCI, points	4 (2, 5)	3 (2, 5)	4 (3, 5)	4 (2, 5)	4 (3, 5)	4 (3, 5)	< 0.001
APACHE II, points	16 (12, 20)	14 (11, 18)	16 (12, 20)	16 (12, 21)	18 (14, 22)	21 (16, 26)	< 0.001
SOFA, points	3 (2, 5)	2 (1, 3)	4 (3, 5)	4.5 (3, 6)	5 (4, 6)	5 (4, 7)	< 0.001
VIS max, points	1.2 (0, 25)	0 (0, 0)	10 (9.2, 12.6)	22.5 (20, 25)	39.7 (35, 40)	80 (60, 126.2)	< 0.001
VIS average, points	0 (0, 19.2)	0 (0, 0)	12 (9.3, 15.5)	19.6 (12, 25.3)	30.2 (19.9, 40.1)	55.6 (30.4, 94.8)	< 0.001
Sepsis, n (%)	923 (81.8)	853 (44.9)	239 (64.2)	310 (74.9)	179 (81.0)	384 (83.7)	< 0.001
Heartrate max (bpm)	108 (95, 123)	104 (92, 116)	106 (95, 119.2)	110 (96, 124)	116 (105, 129)	130 (114, 145)	< 0.001
Respirate max (bpm)	24 (20, 29)	23 (20, 28)	24 (20, 28)	24.5 (20, 30)	25 (21, 30)	26 (22, 32)	< 0.001
SBP average (mmHg)	124.9 (116.1, 135.4)	131.4 (121.2, 140.9)	122.3 (115.6, 129.4)	120.9 (114.5, 129.3)	120.1 (114.8, 127)	116.9 (110.2, 125.2)	< 0.001
DBP average (mmHg)	62.4 (56.8, 68)	63.3 (57.6, 69.5)	60.4 (55.6, 65.9)	61.8 (56.4, 67.1)	62.6 (57.2, 67.2)	61.8 (56.1, 67.2)	< 0.001
MAP average (mmHg)	83.5 (77.7, 90.3)	86.1 (79.9, 92.7)	81.1 (76.2, 87.8)	81.3 (76.8, 88.4)	82.5 (77.7, 87.1)	80.8 (74.4, 86.5)	< 0.001
CVP average (mmHg)	6.9 (5.7, 8.5)	6.8 (5.7, 8.4)	6.8 (5.6, 8.4)	7.1 (5.8, 8.8)	6.9 (5.8, 8.1)	7.2 (5.7, 8.9)	0.068
Lactate max (mmol/L)	2.5 (1.6, 4.1)	2.2 (1.3, 3.3)	2.4 (1.7, 3.9)	2.7 (1.8, 4.4)	3 (2.2, 4.4)	4.8 (2.7, 10.2)	< 0.001
Fluid-in (mL)	2421 (1820, 3033)	2134 (1604, 2722)	2474 (2044, 3010)	2583 (2032, 3020)	2784 (2402, 3189)	3353 (2634, 4595)	< 0.001
Fluid-out (mL)	2555 (1750, 3485)	2324 (1560, 3232)	2668 (1903, 3352)	2632 (1950, 3546)	2772 (2154, 3540)	3185 (2188, 4135)	< 0.001
Fluid-balance (mL)	−72.1 (−638, 371)	−113 (−690, 249)	−144 (−651, 344)	−142 (−635, 321)	8 (−511, 734)	323 (−452, 1287)	< 0.001
ICU LOS (days)	2.9 (1.7, 6.1)	2.4 (1.5, 4.9)	2.8 (1.8, 6.0)	3.7 (2.0, 7.5)	3.6 (2.2, 6.8)	5.2 (2.7, 10.4)	< 0.001
Hospital LOS (days)	24 (15, 41)	22 (14, 35)	25 (16, 42)	27 (18, 50)	26 (17, 44)	30 (18, 53)	< 0.001
ICU mortality, n (%)	118 (3.5)	25 (1.3)	9 (2.4)	25 (6.0)	8 (3.6)	51 (11.1)	< 0.001
Hospital mortality, n (%)	269 (8.0)	83 (4.4)	19 (5.1)	47 (11.4)	15 (6.8)	105 (22.9)	< 0.001
28-day mortality, n (%)	421 (12.5)	143 (7.5)	35 (9.4)	50 (12.1)	38 (17.2)	155 (33.8)	< 0.001

Results are reported as mean (95% confidence interval) or number (%)

*BMI* body mass index, *COPD* chronic obstructive pulmonary disease, *CHD* chronic heart failure, *CKD* chronic kidney disease, *CCI* Charlson Comorbidity Index, *APACHE II* Acute Physiology and Chronic Health Evaluation II, *SOFA* sequential organ failure assessment, *VIS* Vasoactive-Inotropic Score, *SBP* systolic blood pressure, *DBP* diastolic blood pressure, *MAP* mean arterial pressure, *CVP* central venous pressure, *ICU* intensive care unit, *LOS* length of stay

Stratified analysis revealed distinct distribution characteristics of VIS across different surgical types (Table 2). Specifically, the vast majority of patients undergoing head and neck surgery, vascular surgery, and orthopedic surgery required no vasoactive agents or only low-dose vasoactive agent administration postoperatively. In contrast, among patients undergoing gastrointestinal or hepatobiliary surgeries, which are characterized by high infectious risk and complex surgical procedures, the proportion of patients with a VIS ≥ 45 points reached 20%. An elevated VIS strongly indicates the occurrence or aggravation of post-surgical infection. Table 2 shows the infection characteristics of post-noncardiac surgical patients. Among patients admitted to the ICU after noncardiac surgery, 58.4% met the Sepsis 3.0 criteria for sepsis within the first 24 h. The prevalence of sepsis in post-noncardiac surgical patients with a VIS of 0–5 was 44.9%, and it exhibited a corresponding upward trend with increasing VIS, ultimately reaching 83.7% in the patient subgroup with a VIS exceeding 45. Consistently, infection-related biomarkers, including Procalcitonin (PCT) and C-reactive protein (CRP), displayed an identical trend of elevation with higher VIS. Among all postoperative infections with confirmed anatomical sites, pulmonary infection accounted for the largest proportion, followed sequentially by bloodstream infection, urinary tract infection and abdominal infection. Regarding the distribution of infections across the five VIS groups, no significant intergroup difference was observed in the incidence of skin infection. In contrast, for the remaining infection types, patients with higher VIS exhibited a significantly increased likelihood of developing an infection.

**Table 2:** Infection indicators, surgery sites and positive culture detection of study population

	Total (*n* = 3365)	0–5 (*n* = 1899)	6–15 (*n* = 372)	16–30 (*n* = 414)	31–45 (*n* = 221)	> 45 (*n* = 459)	p
Sepsis, n (%)	1965 (58.4)	853 (44.9)	239 (64.2)	310 (74.9)	179 (81.0)	384 (83.7)	< 0.001
PCT (ng/mL)	1.6 (0.3, 7.3)	0.7 (0.2, 2.9)	2.3 (0.6, 8.5)	3.3 (0.9, 9.4)	3.5 (1.3, 16.7)	11.1 (3.0, 31.6)	< 0.001
CRP (mg/mL)	73 (31, 134)	60 (23, 116)	74 (33, 128)	84 (45, 164)	88 (41, 150)	106 (50, 168)	< 0.001
Surgery sites, n (%)							< 0.001
Head and Neck	380 (11.3)	326 (17.2)	23 (6.2)	21 (5.1)	4 (1.8)	6 (1.3)	
Hepatobiliary	719 (21.4)	293 (15.4)	93 (25.0)	111 (26.8)	71 (32.1)	151 (32.9)	
Gastrointestinal	624 (18.5)	257 (13.5)	85 (22.8)	79 (19.1)	75 (33.9)	128 (27.9)	
Vascular	319 (9.5)	196 (10.3)	36 (9.7)	40 (9.7)	18 (8.1)	29 (6.3)	
Orthopedic	195 (5.8)	106 (5.6)	27 (7.3)	34 (8.2)	9 (4.1)	19 (4.1)	
Gynecological	76 (2.3)	47 (2.5)	5 (1.3)	14 (3.4)	3 (1.4)	7 (1.5)	
Urological	64 (1.9)	34 (1.8)	11 (3.0)	5 (1.2)	4 (1.8)	10 (2.2)	
Others	571 (17.0)	340 (17.9)	55 (14.8)	74 (17.9)	28 (12.7)	74 (16.1)	
Positive culture, n (%)	1309 (53.7)	555 (46.2)	156 (57.1)	196 (55.8)	116 (60.7)	286 (68.1)	< 0.001
Respiratory, n (%)	736 (30.2)	298 (24.8)	78 (28.6)	115 (32.8)	47 (24.6)	198 (47.1)	< 0.001
Blood, n (%)	253 (10.4)	82 (6.8)	26 (9.5)	43 (12.3)	21 (11.0)	81 (19.3)	< 0.001
Urine, n (%)	191 (7.8)	74 (6.2)	26 (9.5)	36 (10.3)	9 (4.7)	46 (11.0)	0.002
Peritoneal, n (%)	119 (4.9)	41 (3.4)	10 (3.7)	20 (5.7)	19 (9.9)	29 (6.9)	< 0.001
Skin, n (%)	66 (2.7)	32 (2.7)	4 (1.5)	6 (1.7)	8 (4.2)	16 (3.8)	0.167
Others, n (%)	700 (20.8)	309 (25.7)	90 (33.0)	97 (27.6)	64 (33.5)	140 (33.3)	0.006

Results are reported as mean (95% confidence interval) or number (%)

*PCT* Procalcitonin, *CRP* C-reactive protein

**Fig. 2:**
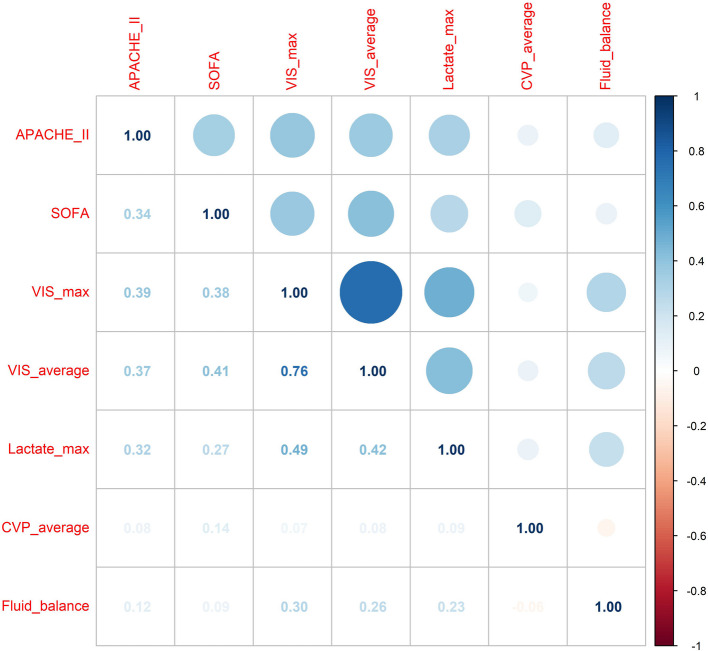
VIS correlation analysis heatmap

Pearson correlation analysis results for VIS and other key prognostic indicators are illustrated in Fig. 2. Despite the observation that patients in the high VIS group presented with notably higher APACHE II scores and SOFA scores, VIS exhibited only a moderate positive correlation with these two scoring systems. Specifically, the Pearson correlation coefficient (r) between the APACHE II score and the maximum VIS documented within the first 24 h postoperatively was calculated as 0.39. Correspondingly, the Pearson correlation coefficient between the SOFA score and the 24-h maximum VIS was 0.38, indicating a moderate positive correlation in both cases. In contrast, the blood lactate level displayed a slightly stronger positive correlation with maximum and average VIS (r = 0.49, 0.42, respectively). Conversely, the correlations between the VIS and central venous pressure (CVP) as well as the 24-h total fluid balance were found to be weak, with correlation coefficients not indicative of a meaningful linear association.

**Fig. 3:**
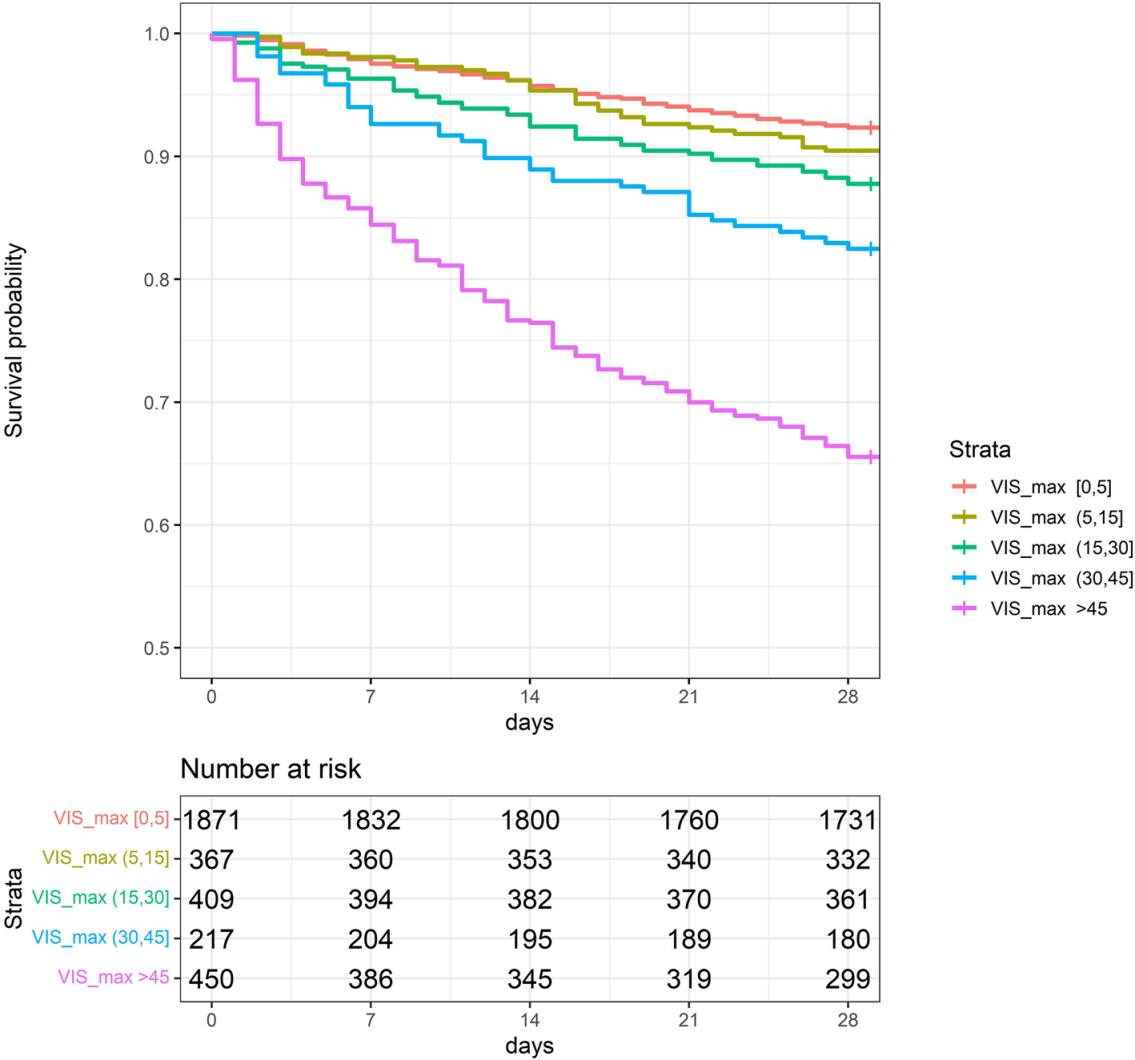
Kaplan–Meier Survival Curves by Different VIS Groups

Kaplan–Meier survival curves for the five VIS groups are shown in Fig. 3. A statistically significant difference in survival rates was observed among the five groups (*P* < 0.05), with patients having higher VIS exhibiting significantly poorer prognostic outcomes. Notably, the survival rate of patients with a VIS > 45 points was markedly lower than that of patients in all other VIS subgroups.

Supplementary Table 1 presents the variables with significant differences between the 28-day survival and mortality groups. After including variables with statistical significance in univariate analysis and clinical relevance, a stepwise logistic regression analysis was performed for the VIS to predict 28-day mortality (Table 3). After adjusting for potential confounding factors (age, gender, CCI, APACHE II score, sepsis occurrence, maximum serum lactate level), a 24-h maximum VIS > 45 points was identified as an independent predictor of 28-day mortality in the study population (OR 2.126, 95% CI: 1.536–2.941, *P* < 0.001). The final multivariate model included 11 predictive variables, with an events per variable (EPV) of 42 (421 outcome events), which exceeded the recommended threshold for model stability. Multicollinearity assessment showed VIF values of all variables were < 5, indicating no significant multicollinearity. Calibration analysis confirmed good calibration of the multivariate model (Supplemental Fig. 1).

**Table 3:** The multivariate logistics regression model for 28-day mortality of study patients

Variables	OR	Lower CI	Upper CI	P
Male	1.124	0.876	1.442	0.360
Age	1.002	0.993	1.011	0.691
CCI	1.102	1.022	1.188	0.012
APACHE II	1.086	1.066	1.105	< 0.001
VIS max (0,5)	Reference			
VIS max (5,15)	0.929	0.618	1.396	0.723
VIS max (15,30)	1.128	0.779	1.634	0.523
VIS max (30,45)	1.498	0.978	2.294	0.063
VIS max > 45	2.126	1.536	2.941	< 0.001
Sepsis	0.864	0.667	1.118	0.266
Lactate max	1.144	1.109	1.180	< 0.001

*OR* odds ratio, *CI* confidence interval, *BMI* body mass index, *CCI* Charlson Comorbidity Index, *APACHE II* Acute Physiology and Chronic Health Evaluation II, *VIS* Vasoactive-Inotropic Score, *ICU* intensive care unit

**Fig. 4:**
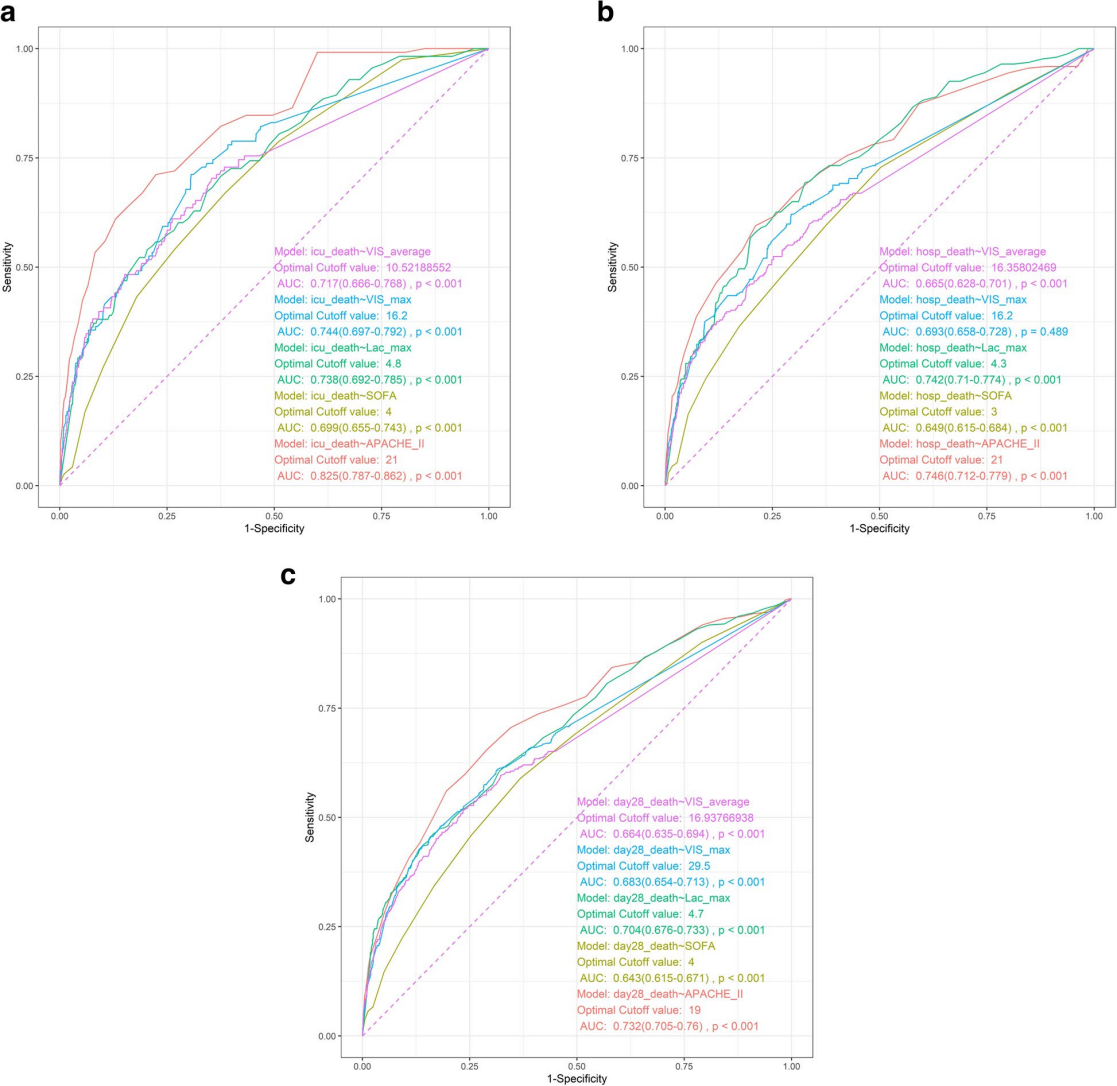
**a** ROC Curve of VIS for Predicting ICU Mortality. **b** ROC Curve of VIS for Predicting hospital mortality. **c** ROC Curve of VIS for Predicting 28-day mortality

ROC curve analysis was conducted to compare the predictive performance of different clinical scoring systems and biochemical indicators (Fig. 4a–4c). In the prediction of ICU mortality, the area under the ROC curve (AUC) for the APACHE II score was 0.825, while the AUC values for the SOFA score, blood lactate level and 24-h maximum VIS were 0.688, 0.738 and 0.744, respectively (Fig. 4a). These findings demonstrate that the VIS has a superior predictive value for ICU mortality in post-noncardiac surgical patients compared with the SOFA score and blood lactate level, although it is slightly inferior to the APACHE II score. With regard to long-term prognostic assessment, the AUC of the VIS for predicting in-hospital mortality was 0.693, and its AUC for predicting 28-day mortality was 0.683 (Fig. 4b, 4c), indicating a moderate predictive ability for long-term outcomes.

Internal validation via the bootstrap method with 1000-resamplings showed that the overall shrinkage factors of the VIS-based predictive models for ICU mortality, in-hospital mortality, and 28-day mortality were 0.9996, 0.9301, and 0.9184, respectively (all > 0.85) (Supplementary Fig. 2). These findings indicated that the VIS prognostic models had no significant overfitting and favorable coefficient stability, thus obviating the need for shrinkage correction. Further subgroup analyses revealed that the cut-off values of the VIS varied to a certain extent among patients with different disease severities (Supplementary Fig. 3). The VIS yielded superior predictive performance with a lower cut-off value in more critically ill patients, such as those with an APACHE II score ≥ 16.

## Discussions

This retrospective cohort study demonstrated that an elevated Vasoactive-Inotropic Score constitutes an independent predictor of mortality in post-noncardiac surgical patients admitted to the ICU. The admission and management of critically ill post-surgical patients represent a core mandate of the ICU. Notably, the majority of post-noncardiac surgical patients admitted to the ICU do not require therapeutic intervention with vasoactive agents. The administration of vasoactive agents or inotropic drugs in this patient population is often indicative of the development of complications or infectious processes [21].

The VIS is a well-established and widely utilized tool for the quantitative assessment of cumulative vasoactive agent dosages. A large body of clinical evidence has consistently validated its strong predictive utility for adverse prognostic outcomes in ICU patients. Gaies and colleagues conducted an analysis to explore the association between VIS and clinical outcomes in infants undergoing cardiac surgery, and their findings confirmed that the maximum VIS value within the first 48 h postoperatively was significantly correlated with the occurrence of adverse events9. In another study, Kwon et al. reported that the VIS measured within 48 h after on-pump coronary artery bypass grafting (CABG) exhibited a significant positive association with long-term all-cause mortality [22]. Additionally, Zhang et al. demonstrated in their research that the maximum VIS within the initial 24 h post-admission was a reliable predictor of adverse clinical endpoints [23]. Crow et al. further verified that a VIS ≥ 3 was independently associated with an increased risk of composite adverse outcomes following cardiac surgery [24].

This study enrolled all post-noncardiac surgical patients admitted to ICU, encompassing those who underwent gastrointestinal surgery, hepatobiliary surgery, orthopedic surgery, vascular surgery, gynecological surgery, urological surgery, and other surgical procedures. The results revealed that more than half of post-noncardiac surgical ICU-admitted patients required no vasoactive agent therapy or only received low-dose vasoactive agent treatment. With the elevation of the VIS, the clinical prognosis of patients deteriorated significantly, accompanied by a gradual increase in ICU mortality, in-hospital mortality and 28-day all-cause mortality. Survival curve analysis further demonstrated that the survival rate of patients with a VIS > 45 was significantly lower than that of patients in other VIS subgroups.

The primary risk factor contributing to the increased dosage of vasoactive drugs in post-noncardiac surgical patients is the development of infection [25]. Post-noncardiac surgical patients often exhibit impaired tissue integrity and compromised organ function, rendering them susceptible to endotoxemia and intestinal flora translocation [26–28]. These pathological processes can subsequently progress to septic shock, which is characterized by abnormal vascular permeability and disturbed vascular tone. Currently, fluid resuscitation combined with vasoactive agent administration constitutes one of the first-line therapeutic strategies for patients with septic shock14, and the dosage of vasoactive drugs is typically positively correlated with the severity of the patient’s clinical condition. Notably, the Sequential Organ Failure Assessment (SOFA) score, an established diagnostic criterion for sepsis, incorporates the dosage of vasoactive drugs as one of the key parameters for evaluating cardiovascular function [29]. An elevation in the VIS is strongly suggestive of the presence of infection. For patients undergoing non-clean area surgeries (e.g., gastrointestinal and hepatobiliary surgery), the likelihood of postoperative VIS elevation was significantly increased. Post-noncardiac surgical patients admitted to the ICU are at an extremely high risk of developing sepsis. Among patients with microbiologically confirmed infection, pulmonary infection is the most prevalent, followed by bloodstream infection. As the VIS increases, the likelihood of sepsis and infection rises significantly.

Correlation analysis revealed that the VIS exhibited a weak correlation with clinical indicators including the APACHE II score, SOFA score, blood lactate level, central venous pressure and 24-h total fluid balance. This finding implies that the VIS possesses relatively independent predictive utility, which was further validated by logistic regression analysis. The regression model demonstrated that a VIS > 45 was an independent predictor of 28-day all-cause mortality. Comparative analysis of different predictive models indicated that the VIS had superior predictive performance for ICU mortality in post-noncardiac surgical patients compared with the SOFA score and blood lactate level, with an AUC of 0.744 and an optimal cut-off value of 16.2, which is consistent with the findings of previous studies [30]. However, in terms of predicting in-hospital mortality and 28-day all-cause mortality, the predictive efficacy of the VIS declined, and its AUC was lower than that of the blood lactate level.

Although the predictive performance of VIS was slightly inferior to that of the APACHE II in terms of AUC, the APACHE II and SOFA scores incorporate a broader range of indicators, whereas the VIS achieves considerable predictive efficacy with a single metric. Furthermore, due to the inherent limitations of the APACHE II and SOFA scores in their design for vasoactive agent dosage assessment—these scores only account for the use of a single vasoactive agent and fail to quantitatively evaluate vasoactive agent burden-the VIS offers distinct clinical advantages. It is readily obtainable and can be dynamically calculated and monitored via critical care medication administration systems in clinical practice. Notably, the VIS demonstrates higher specificity for patients with infection and sepsis. Correlation analyses further confirmed that the VIS exerts an independent predictive effect from the aforementioned scoring systems, thus complementing their deficiencies.

The predictive performance of VIS for 28-day mortality was attenuated, which may be attributed to the stronger correlation between vasoactive agent dosage administered during ICU stay and in-hospital mortality risk. For patients receiving vasoactive agents, hemodynamic stability is critically dependent on adjustments to vasoactive agent dosages, and a slight increase in dosage may indicate disease progression, thus resulting in a higher AUC value for mortality prediction during ICU admission. After discharge from the ICU, most patients had discontinued vasoactive agent administration following ICU management and further stratification of mortality risk. Their outcomes were therefore influenced by other mortality risk factors, including underlying diseases, surgical recovery status and postoperative complications, which consequently diminished the predictive value of VIS and led to a concomitant elevation of its optimal predictive cut-off value.

Given that the VIS can reflect the short-term stability of hemodynamics and cardiovascular function, while blood lactate levels are indicative of persistent systemic tissue hypoxia. The VIS may be more valuable for evaluating the short-term disease severity. In contrast, blood lactate levels can predict long-term prognosis by reflecting hypoxic-related organ dysfunction. Future studies may focus on the combined application of the VIS and blood lactate levels to enhance the accuracy of prognostic prediction in post-noncardiac surgical patients.

This study has several limitations. First, the VIS did not demonstrate superior discriminative ability for mortality risk compared with well-established scoring systems such as the APACHE II score, thus precluding its use as a standalone risk stratification tool. Second, as an observational study, the present research requires more prospective study designs to verify whether VIS guided interventions can improve patient outcomes, which would facilitate the clinical implementation of the VIS. Third, the optimal VIS cut-off values varied to a certain extent across different outcome measures, and subgroup analyses suggested that this variation might be attributable to differences in patient disease severity, which increases the complexity of its clinical application to some degree. Fourth, this was a single-center study conducted in a Chinese population with internal validation performed; additional external validation is therefore needed to confirm the robustness of the VIS when extrapolated to other medical centers. Fifth, other surgery-related influencing factors, such as emergency surgery status, anesthesia duration, and anesthetic agent use, were not investigated in this study, and further research is required to evaluate the impact of these factors. Finally, multiple statistical comparisons were performed without multiplicity adjustment of P values in this study, which may increase the risk of Type I error.

## Conclusion

Adult patients admitted to the ICU following noncardiac surgical procedures exhibit a notably high incidence of infection and sepsis, coupled with poor clinical outcomes. A higher VIS strongly indicates adverse postoperative infection and poor short-term survival outcomes. In our Chinese surgical ICU cohort, the VIS demonstrates strong and consistent independent prognostic value via internal validation, especially for ICU mortality. While it has not outperformed these well-established tools such as APACHE II and SOFA, it can function as a readily accessible and complementary indicator to existing scoring systems and deliver additional real-time insights for hemodynamic assessment. Whether VIS-guided interventions translate to improved patient outcomes merits further investigation in larger prospective studies.

## Supplementary Information

Supplementary Material 1.

Supplementary Material 2.

Supplementary Material 3.

Supplementary Material 4.

Supplementary Material 5.

Supplementary Material 6.

## Data Availability

The datasets used and analyzed during the current study are available from the corresponding author on reasonable request.

## Abbreviations

VIS: Vasoactive-Inotropic Score
ICU: Intensive care unit
APACHE II: Acute Physiology and Chronic Health Evaluation II
SOFA: Sequential Organ Failure Assessment
OR: Odds ratio
CI: Confidence interval
CCI: Charlson Comorbidity Index
LOS: Length of stay
PCT: Procalcitonin
CRP: C-reactive protein
BMI: Body mass index
COPD: Chronic obstructive pulmonary disease
CHD: Coronary heart disease
CKD: Chronic kidney disease
