# Supplementary figures and images for: Assessment of the vasoactive-inotropic score in prognostic evaluation of critical patients following noncardiac surgery: a retrospective, observational study
Source: BMC Anesthesiol. 2026 Mar 9;26:239. doi: 10.1186/s12871-026-03724-z (PMC13085579; doi:10.1186/s12871-026-03724-z)

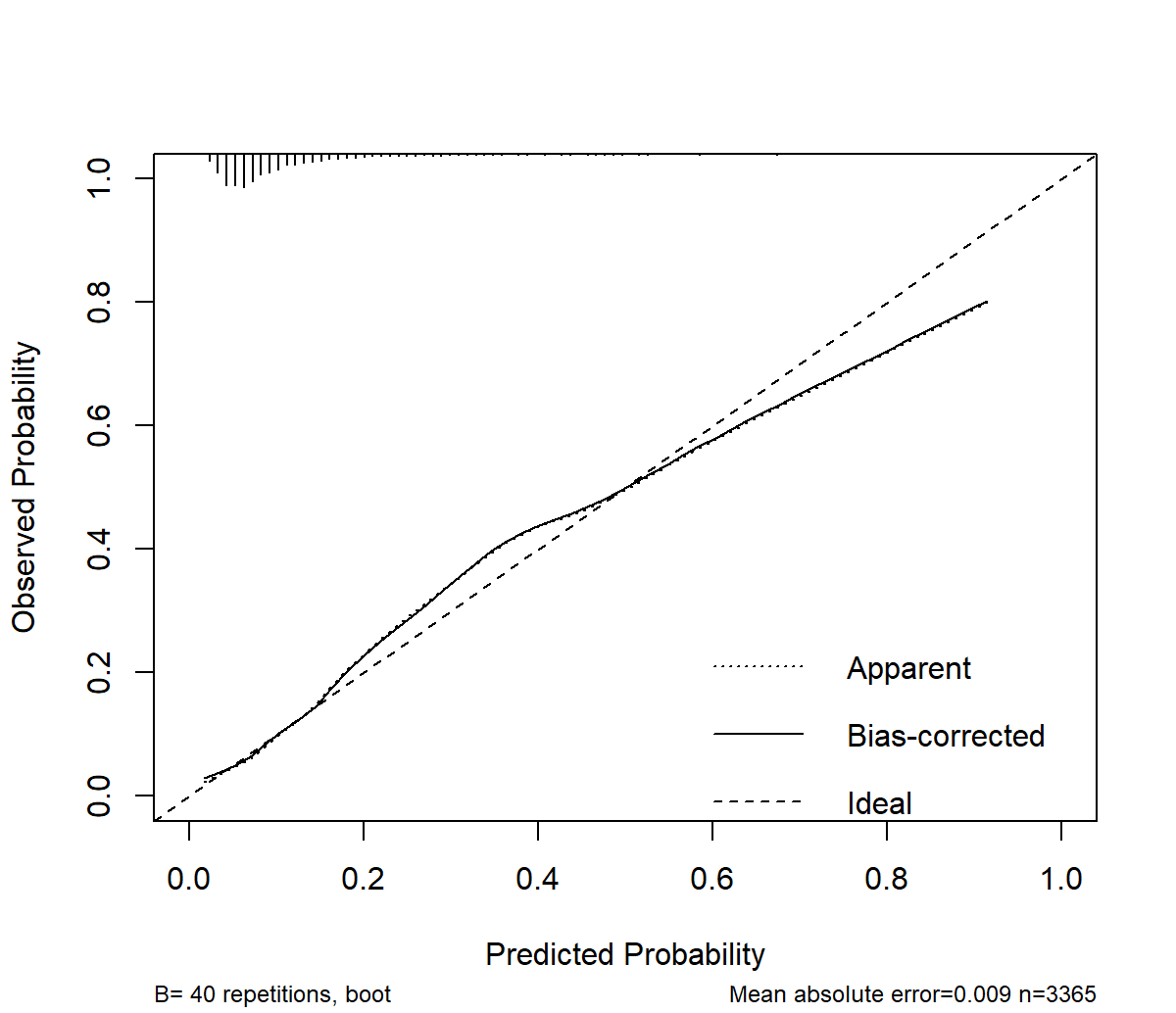

Supplement: Supplementary file 1 — Supplementary Material 1. [file 12871_2026_3724_MOESM1_ESM.png]

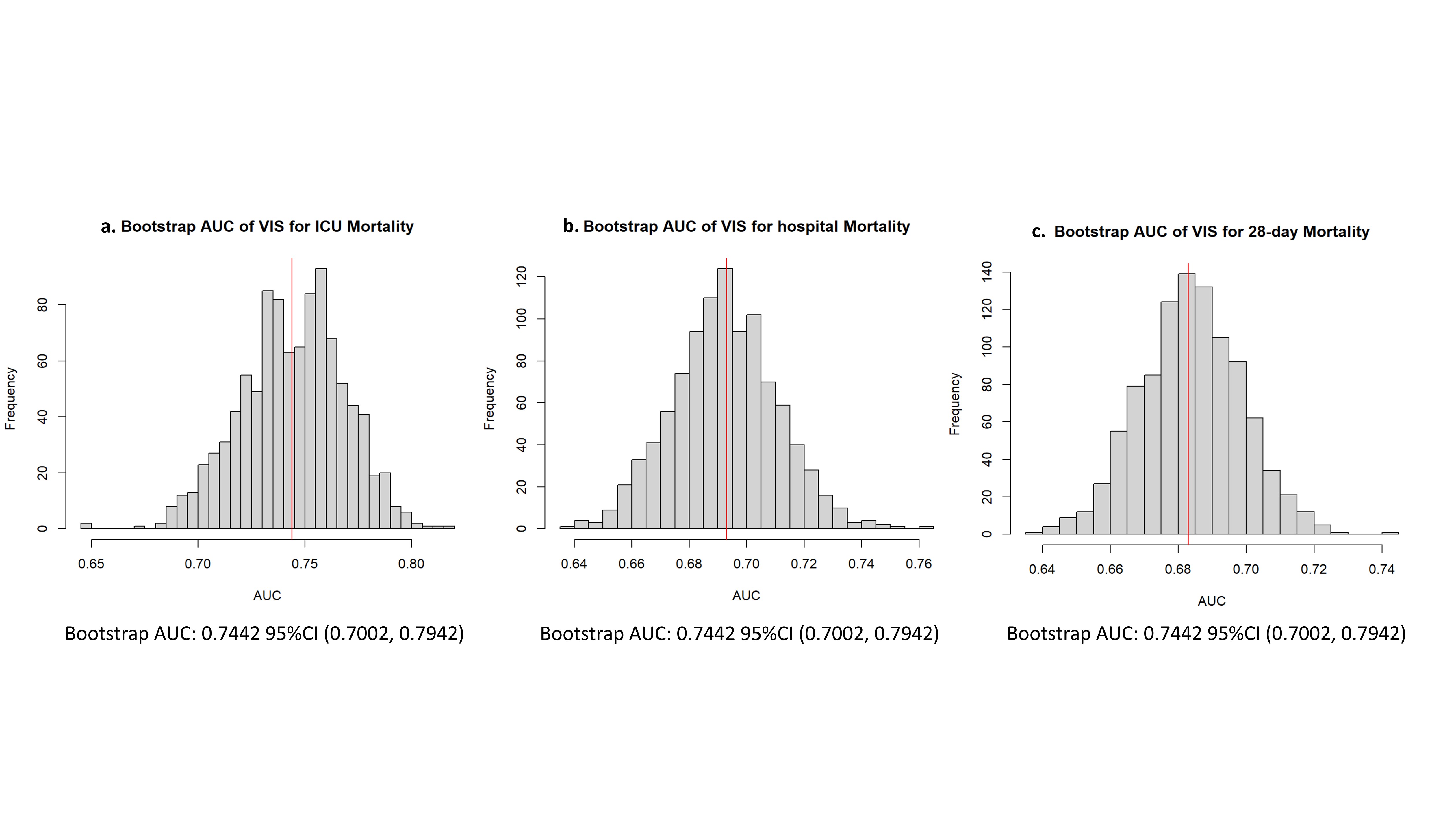

Supplement: Supplementary file 2 — Supplementary Material 2. [file 12871_2026_3724_MOESM2_ESM.jpg]

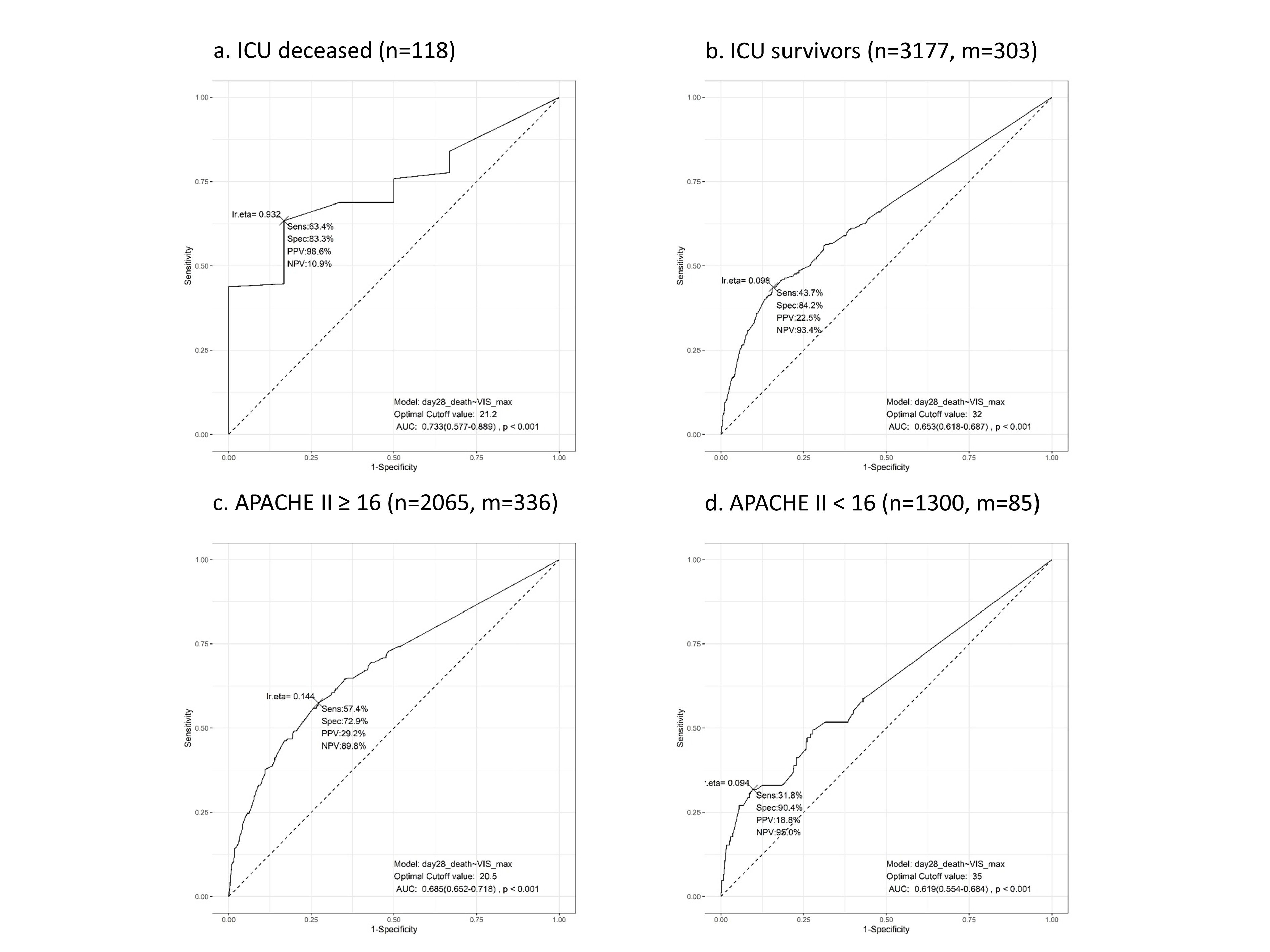

Supplement: Supplementary file 3 — Supplementary Material 3. [file 12871_2026_3724_MOESM3_ESM.jpg]
